# 2518. Lead optimization on 26-membered thiopeptide antibiotics and identification of pre-clinical candidates against *Clostridioides difficile* and *Staphylococcus aureus* for impetigo

**DOI:** 10.1093/ofid/ofad500.2136

**Published:** 2023-11-27

**Authors:** Hee-Jong Hwang, Young-Jin Son, Dahyun Kim, Jusuk Lee, Clovis Shyaka, Jin-Hwan Kwak, Hyunjoo Pai, Mina Rho, Jong-Hwan Park, Young-Rok Kim, Sungji Jung

**Affiliations:** A&J Science, Daegu, Taegu-jikhalsi, Republic of Korea; A&J Science, Daegu, Taegu-jikhalsi, Republic of Korea; A&J Science, Daegu, Taegu-jikhalsi, Republic of Korea; A&J Science, Daegu, Taegu-jikhalsi, Republic of Korea; A&J Science, Daegu, Taegu-jikhalsi, Republic of Korea; Handong Glodal University, Pohang, Kyongsang-bukto, Republic of Korea; Department of Internal Medicine, Hanyang University College of Medicine, Seongdong-gu, Seoul-t'ukpyolsi, Republic of Korea; Hanyang University, Seoul, Seoul-t'ukpyolsi, Republic of Korea; Chonnam National University, Kwangju, Kwangju-jikhalsi, Republic of Korea; Handong Glodal University, Pohang, Kyongsang-bukto, Republic of Korea; Handong Glodal University, Pohang, Kyongsang-bukto, Republic of Korea

## Abstract

**Background:**

Thiopeptides are structurally complex natural products that exert potent antimicrobial activity against Gram-positive pathogens by inhibiting bacterial protein synthesis. However, there has not been any attempt to conduct extensive medicinal chemistry campaign on these natural products, due to their chemical complexity. The advent of efficient syntheses of thiopeptides led us to identify promising pre-clinical candidates: AJ-024 against *Clostridioides difficile* and AJ-147 against *Staphylococcus aureus* for the indication of impetigo.

**Methods:**

AJ-024 was screened against extensively classified *C. difficile* clinical isolates. Its time-kill kinetics and *in vivo* mouse efficacy were investigated against hypervirulent *C. difficile* ribotype 027. Metagenomic sequencing (16S rRNA) was performed to examine AJ-024's impact on gut microbiome.

Similarly, AJ-147 was screened against methicillin-resistant *S. aureus* clinical isolates. Its time-kill kinetics, as well as its *in vivo* SSTI model were investigated. Pro-inflammatory cytokines were measured.

Pharmacokinetic studies of these two agents, as well as their ADME properties and toxicity have also been investigated.

**Results:**

AJ-024 has potent antibacterial activity, along with rapid bactericidal action against *C. difficile* ribotype 027 resulting in 99.99% reduction in 4X MIC in 3 hours. No recurrence was observed in the mouse *in vivo* model and this was corroborated by our 16S rRNA sequencing data. AJ-024 exerts minimal impact on beneficial gut-microbiome.

AJ-147 exhibited a potent antibacterial activity against mupirocin resistant *S. aureus*. *In vivo* efficacy in a SSTI model revealed that AJ-147 is x1,000 more active than mupirocin. AJ-147 downregulates pro-inflammatory cytokines, such as IL-1*ß* and IL-6, that might elicit beneficial host immune response.

Impact of AJ-024 on gut microbiome

**Figure:**
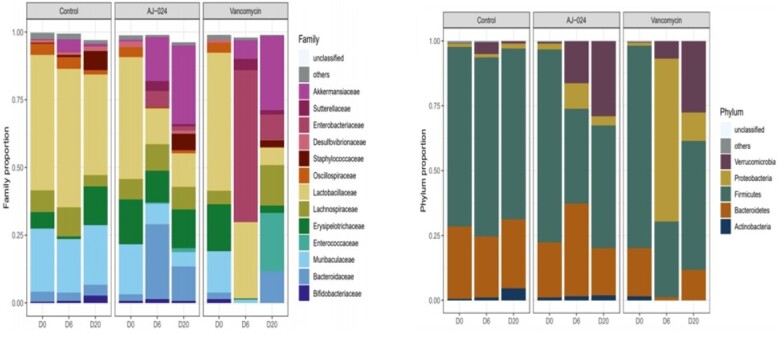

Investigation of AJ-024's impact on gut microbiome through 16s rRNA sequencing

In vivo efficacy of AJ-147 in SSTI model

**Figure:**
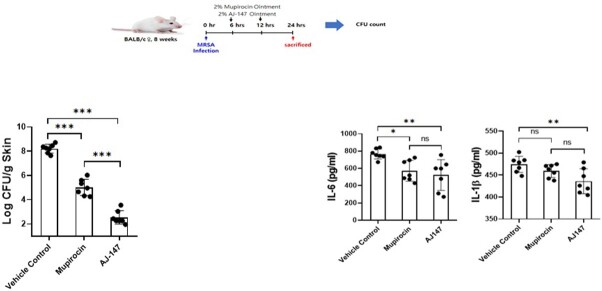

AJ-147 compares favorably to mupirocin and downregulates pro-inflammatory cytokines

**Conclusion:**

Our medicinal chemistry campaign on thiopeptide antibiotics have led us to identify two preclinical candidates, AJ-024 and AJ-147. These two agents possess distinctive advantages compared to the first-line of treatments for each indication. Efforts are being directed to the completion of IND-enabling studies.

**Disclosures:**

**Hee-Jong Hwang, PhD**, A&J Science: Stocks/Bonds|KHIDI: Grant/Research Support **Young-Jin Son**, A&J Science: Employee of A&J Science|A&J Science: Ownership Interest|KHIDI: Grant/Research Support **Dahyun Kim, n/a**, A&J Science: Employee of A&J Science **Jusuk Lee, Ph.D.**, A&J Science: Employee of A&J Science **Clovis Shyaka, n/a**, A&J Science: Employee of A&J Science

